# Spinal dI4 Interneuron Differentiation From Human Pluripotent Stem Cells

**DOI:** 10.3389/fnmol.2022.845875

**Published:** 2022-04-08

**Authors:** Jia Xu, Liang-Jiang Huang, Zhengyu Fang, Hong-Mei Luo, Yun-Qiang Chen, Ya-Jie Li, Chen-Zi Gong, Hong Chen

**Affiliations:** ^1^Department of Rehabilitation, Tongji Hospital, Tongji Medical College, Huazhong University of Science and Technology, Wuhan, China; ^2^Stem Cell Research Center, Tongji Hospital, Tongji Medical College, Huazhong University of Science and Technology, Wuhan, China; ^3^Department of Neurological Rehabilitation, Taihe Hospital, Hubei University of Medicine, Shiyan, China

**Keywords:** spinal cord, interneuron, human pluripotent stem cells, GABA, differentiate

## Abstract

Spinal interneurons (INs) form intricate local networks in the spinal cord and regulate not only the ascending and descending nerve transduction but also the central pattern generator function. They are therefore potential therapeutic targets in spinal cord injury and diseases. In this study, we devised a reproducible protocol to differentiate human pluripotent stem cells (hPSCs) from enriched spinal dI4 inhibitory GABAergic INs. The protocol is designed based on developmental principles and optimized by using small molecules to maximize its reproducibility. The protocol comprises induction of neuroepithelia, patterning of neuroepithelia to dorsal spinal progenitors, expansion of the progenitors in suspension, and finally differentiation into mature neurons. In particular, we employed both morphogen activators and inhibitors to restrict or “squeeze” the progenitor fate during the stage of neural patterning. We use retinoic acid (RA) which ventralizes cells up to the mid-dorsal region, with cyclopamine (CYC), an SHH inhibitor, to antagonize the ventralization effect of RA, yielding highly enriched dI4 progenitors (90% Ptf1a^+^, 90.7% Ascl1^+^). The ability to generate enriched spinal dI4 GABAergicINs will likely facilitate the study of human spinal IN development and regenerative therapies for traumatic injuries and diseases of the spinal cord.

## Introduction

The spinal cord relays sensory inputs from the periphery to the higher-order centers in the brain while carrying signals from the brain to the periphery to control movement and regulate autonomic functions. These relaying functions are mediated and/or regulated by an array of interneurons (INs) within the spinal cord. These INs also form networks and function as local executive units to generate neural oscillations and subsequent rhythmic motor activity, termed as locomotor central pattern generators (CPGs; Goulding and Pfaff, 2005; Grillner, 2006). The main inhibitory interneurons participating in CPG functions are dI4 GABAergic INs. They are located in the dorsal horn (laminaeIII and VI) of the spinal cord, receiving and decoding peripheral somatosensory inputs and projecting ipsilaterally to proprioceptive terminals to convey somatosensory information (Alaynick et al., 2011). These GABAergic INs also form inhibitory synapses with glutamatergic proprioceptive Ia afferent terminals near motor neurons, regulating CPG function and the sensory-motor coupling (Vallstedt and Kullander, 2013).

**Figure 1:**
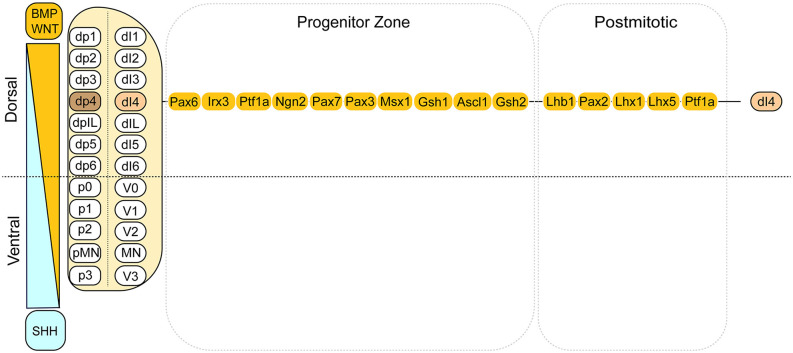
Summary of the dorsal domains and the transcription factors associated with the dI4 INs. This cartoon illustrates the dorsal domains and the corresponding cell types derived. On the left side, it depicts the dorsally derived BMP and WNT gradient. On the right side, the transcription factors are listed for dI4 progenitors and post-mitotic neurons, respectively. Adapted from Alaynick et al. (2011) and Lu et al. (2015).

Developmentally, dI4 INs are derived from the dorsal spinal cord, respectively (Zholudeva et al., 2021). The dorsal domains in the developing neural tube are defined by the expression of combined transcription factors (TFs), which in turn is controlled by morphogen gradients of dorsally derived WNTs and/or bone morphogenetic proteins (BMP) and ventrally derived sonic hedgehog (SHH). Within the idealized segment of the spinal cord, these morphogen gradients establish 12 progenitor domains that give rise to seven dorsal IN progenitor domains, pd1–6, and pdIL; four ventral INs progenitor domains, p0–3; and one motor neuron progenitor domain, pMN. The progression from neural progenitor cells to postmitotic neurons spanning embryonic is shown from left to right, although some events are not strictly linear (Alaynick et al., 2011; Lu et al., 2015; Figure 1). Through a series of cell divisions, 22 types of neurons are produced, including eight dorsal INs, 13 ventral INs, and connected motor neurons (Gross et al., 2002; Caspary and Anderson, 2003; Helms and Johnson, 2003; Borowska et al., 2013). The inhibitory INs in the spinal cord are mainly derived from the Ptf1a-expressing progenitors in the dp4 domain. They become dI4 GABAergic INs mediating somatosensory information (Gwak and Hulsebosch, 2011). These developmental insights form the basis of generating enriched populations of spinal INs.

Diseases or injury to the spinal cord sever the connection between the brain and peripheral tissues and disrupt the networks within the spinal cord (McDonald and Sadowsky, 2002; O’Shea et al., 2017; Courtine and Sofroniew, 2019). There are currently no effective therapies to repair the injured spinal cord. Transplantation of neural progenitors has been explored experimentally to induce regeneration from endogenous neurons or to bridge the gap between severed spinal cords. Increasing evidence points to the necessity of spinal but not brain progenitors to achieve therapeutic outcomes (Kadoya et al., 2016). There is therefore a need to generate enriched spinal IN progenitors. Human pluripotent stem cells (hPSCs), including embryonic stem cells (ESCs) and induced pluripotent stem cells (iPSCs), are a promising source of human neuronal lineages, including spinal INs. Possible roles for dI4 interneurons for CPG coordination remain to be determined; notably, however, a cohort of dI4 neurons have been reported to form contacts on Ia afferent terminals near MNs (Betley et al., 2009). Here, we present a detailed monolayer culture protocol on how to derive enriched spinal dI4 GABAergicINs from hPSCs.

## Materials and Equipment

### Biological Materials

• Human Pluripotent Stem Cells (hPSCs). hESC (H9, WA09), and iPSC (IMR-90) cell lines were obtained from WiCell Research Institute (Madison, WI, USA). We anticipate that this protocol could also be applied to other hESC and hiPSC lines.
• Irradiated mouse embryonic fibroblast (MEFs). All the hPSCs were cultured on MEFs as described in the standard protocol^1^.
• Human spinal cord tissues were obtained from aborted human fetal (12 weeks) tissues. The study was approved by the Ethics Committee of Tongji Hospital, Tongji Medical College, Huazhong University of Science and Technology.

### Reagents

• DMEM High Glucose Medium (Hyclone) SH30022.01
• DMEM/F-12 medium (Gibco) 11330032
• Neurobasal Medium (Gibco) 21103049
• Basic fibroblast growth factor (bFGF) (Peprotech) 100–18B
• B27 (without vitamins, 50%) (Gibco) 12587010
• Non-essential amino acid solution (NEAA, 100×) (Gibco) 11140050
• L-Glutamine Additive (GlutaMax-I supplement, 100×) (Gibco) 35050061
• Fetal bovine serum (Gibco) 10091–148
• Serum replacement KSR (Gibco) A3181502
• N-2 Supplement (100×) (Gibco) 17502048
• B-mercaptoethanol (Sigma) M-3148

**Caution**: This substance is toxic, so avoid direct exposure or inhalation.

• CHIR99021 (Tocrisbioscience) 4423/10
• SB431542 (Stemgent) S1067
• DMH1 (Selleckchem) S7146
• Retinoic acid (RA) (Merck) R2625
• Cyclopamine (CYC) (Enzo) BML-GR334–0001
• Brain-derived neurotrophic factor (BDNF) (Peprotech) 450-02
• Glial cell line-derived neurotrophic factor (GDNF) (Peprotech) 450-10
• cAMP (Merck) A6885
• Ascorbic acid (AA) (Merck) A92902
• Y-27632 dihydrochloride (ROCK inhibitor) (Tocrisbioscience) 1254
• Dispase II (Sigma) D4693
• Accutase Solution (Cell Technology) AT104–500
• Dimethyl sulfoxide (DMSO) (Merck) D2650

**Caution**: This substance is toxic. Avoid direct contact or inhalation.

• Gelatin (Thermo Scientific) S006100
• Matrigel (Corning) 354277
• 4% paraformaldehyde (PFA) (Wuhan Haotek Technology) G1101

**Caution**: PFA is toxic and avoid direct contact or inhalation, manipulate it in a fume hood.

• Phosphate buffered saline (PBS) (Hyclone) C10010500BT
• QuickBlock^TM^ Blocking Buffer for Immunol Staining (Beyotime) P0260
• QuickBlock^TM^ Primary Antibody Dilution Buffer for Immunol Staining (Beyotime) P0262
• QuickBlock^TM^ Secondary Antibody Dilution Buffer for Immunofluorescence (Beyotime) P0265
• Fluoromount-G^®^ (Southern Biotech) 0100-01
• Antibodies, refer to Table 1 for details.

**Table 1:** Antibodies and fluorochromes employed in this study.

**Antibody name**	**Brand and item number**	**Species source, dilution ratio**
Sox1	R&D systems, AF3369	Goat, 1:1,000
Hoxb4	Sigma, APREST83782	Rabbit, 1:50
Ptf1a	Santa Cruz, sc-393011	Mouse, 1:50
GABA	Sigma, A2052	Rabbit, 1:500
Lhx1/5	DSHB, 4F2	Mouse, 1:50
NF-200	Sigma, AF5389	Mouse, 1:1,000
MAP2	Abcam, Ab32454	Rabbit, 1:200
NEUN	Millipore, MAB377	Mouse, 1:500
Ascl1	Santa Cruz, sc-374104	Mouse, 1:1,000
Pax2	Biolegend, PRB-276P	Rabbit, 1:1,000
Olig3	Abcam, ab168573	Mouse, 1:100
Cy5	Jackson, 128457	Mouse, 1:250
Alexa Fluor 488	Invitrogen, A21206	Rabbit, 1:1,000
Alexa Fluor 488	Invitrogen, A11055	Goat, 1:1,000
Alexa Fluor 594	Invitrogen, A21203	Mouse, 1:1,000
DAPI	Wuhan Google biology, G1012	0.1 μg/ml

### Equipment

• Plates, 6-well plate/24-well plate (Corning) 3516/3524
• Centrifuge tube (15 ml/50 ml; Jet-Biofil) CFT011150/CFT011500
• T25 flask (Jet-Biofil) TCF011250
• T75 flask (Jet-Biofil) TCF012250
• 2 ml cryotube (Corning) 430659
• Pipette tip (10 μl/200 μl/1 ml; Axygen Corporation) T-300/T-200-Y/T-1000-B
• 0.22 μm disposable PVC filter (Milipore) SLGP033RB
• Confocal microscope (Japan Olympus, FV 3,000)
• Biosafety Cabinet (Thermo Fisher Scientific)
• CO_2_ incubator (Thermo Fisher Scientific)
• Ordinary fluorescence (microscope Olympus, Japan)
• Super clean bench (Beijing Donglian Haar Instrument Manufacturing)
• Low-temperature ultracentrifuge/ordinary tabletop centrifuge (Eppendorf)
• −20°C refrigerator/4°C refrigerator (Qingdao Haier)
• Autoclave (Shanghai Sanshen Medical Devices)
• Electric thermostat blast drying oven (Shanghai Yiheng Scientific Instrument)
• −80°C ultra-low temperature refrigerator (Japan Panasonic)
• Liquid nitrogen tank (Changzhou Zhaosheng)
• X-ray irradiator (Siemens (from Tongji Medical College, Huazhong University of Science and Technology) provided by the affiliated Union Hospital Cancer Hospital)
• Program cooling box Nalgene (Electronic Balance Beijing Sartorius Instrument System)
• Icemaker (Ziegra, Tongji Hospital Geriatrics Laboratory, affiliated toTongji Medical College, Huazhong University of Science and Technology)
• Pipetting gun/Electric pipetting gun (Eppendorf)

### Reagent Setup

**hPSC medium (50 ml)**: For hPSC medium preparation we took 50 ml as an example, and then in a 50 ml centrifuge tube, added 39 ml DMEM/F12, 10 ml KSR, 0.5 ml NEAA, 0.5 ml GlutaMax-I, and 0.35 μl β-mercaptoethanol and mixed it well and stored it in a 4°C refrigerator for no more than 2 weeks. Changing medium: As a principle, the needed amount of medium should be warmed right before use but avoid prolonged warming because many experimental media contain very sensitive components to prolong warming. **CAUTION**: β-mercaptoethanol is considered toxic; can cause nasal and skin irritation, exposure should be avoided, and whenever used, a hood should be used.

**Neural induction medium (NIM, 50 ml)**: Combine 24.5 ml of DMEM/F-12, 24.5 ml of Neurobasal medium, and 0.5 ml of NEAA, 0.5 ml of N-2 supplement into a 50-ml centrifuge tube in a sterile hood. Store the medium at 4°C for up to 2 weeks.

**Neuronal differentiation medium (NDM, 50 ml)**: Combine 49 ml of Neurobasal medium, 0.5 ml of NEAA, and 0.5 ml of N-2 supplement into a 50-ml centrifuge tube in a sterile hood. Store the medium at 4°C for up to 2 weeks.

**Dispase (0.2 mg/ml)**: Prepare a 2 mg/ml solution of Dispase with DMEM/F12, and store at −20°C for up to 2 years. Warm the solution at 37°C for 15 min to dissolve the dispase completely, add DMEM/F12 to a working concentration of 0.2 mg/ml, and store at 4°C for up to 2 weeks.

**DMH1 (10 mM)**: Dissolve 10 mg of DMH1 into 1.3 ml of DMSO and 1.3 ml of ethanol, prepare 50 μl aliquots in sterilized dark tubes and store them at −80°C for up to 6 months.

**SB-431542 (10 mM)**: Dissolve 5 mg into 0.65 ml of DMSO and 0.65 ml of ethanol, prepare 50 μl aliquots in sterilized dark tubes and store them at −80°C for up to 6 months.

**CHIR99021 (10 mM)**: Dilute 10 mg of CHIR99021 in 2.15 ml sterile DMSO. Aliquot 50 μl into 1.5 ml tubes and store at −20°C for up to 3 months.

**Retinoic acid (RA, 100 mM)**: Dilute 50 mg of RA in 1.67ml sterile DMSO. Aliquot 50 μl in 1.5 ml amber tubes and store at −80°C for up to 3 months. To create a 1 mM working solution, dilute 5 μl of stock solution in 495 μl of 100% sterile ethanol. The working solution can be stored at −20°C for up to 2 weeks.

**FGF2 (bFGF, 100 μg/ml)**: Dissolve 25 μg of FGF2 into 250 μl of sterile DPBS with 0.1% (wt/vol) human serum albumin or BSA. Divide the solution into aliquots and store it at −80°C for up to 6 months.

**BDNF, GDNF (100 μg/ml)**: Dissolve 100 μg into 1 ml of sterile DPBS with 0.1% (wt/vol) human serum albumin or BSA. Divide the solution into aliquots and store it at −80°C for up to 6 months.

**cAMP (1 mM)**: Dissolve 4.914 mg of cAMP in 10 ml of sterilized water. Divide the solution into aliquots and store it at −80°C for up to 6 months.

**Y-27632 dihydrochloride (ROCK inhibitor)**: Reconstitute to a concentration of 10 mM in DMSO. Divide into aliquots and store at −20°C. Keep thawed aliquots at 4°C and use them within 1 month.

### Equipment Setup

**Gelatin-coated tissue culture plates**: Distribute 1 ml per well of 0.1% (wt/vol) gelatine solution to 6-well plates. Coat at the incubator for 30 min. Plates with a lid can be stored at 4°C, used within 1 week. Aspirate the coating solution just before use.

**Matrigel-coated tissue culture plates**: Thaw matrigel on ice. Dilute matrigel in DMEM/F-12 (1:50). Distribute 80 μl per well of diluted matrigel to 24-well plates. Store the plates with a lid at 4°C, sealed with parafilm, for up to 1 week. Before use, gently transfer to a 37°C incubator for more than 1 h and aspirate the coating solution while retaining a little.

### Methods

#### Overview of Experimental Design

**Figure 2:**
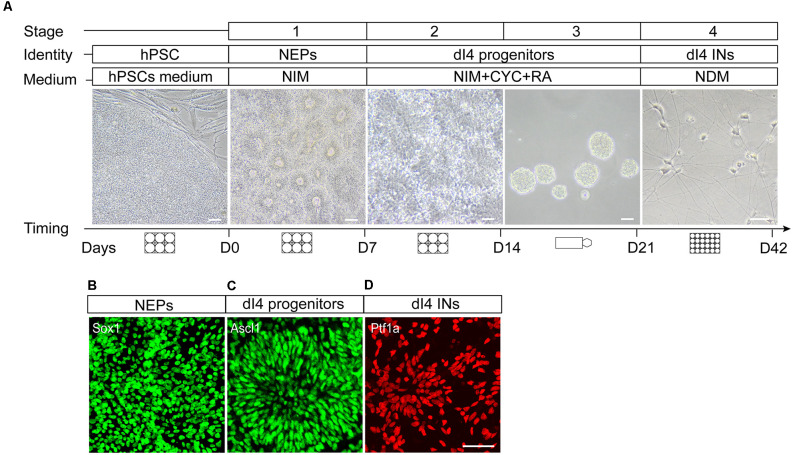
*In vitro* differentiation protocol of spinal dI4 INs from hPSCs. **(A)** Cartoons showing the four stages of the differentiation and corresponding culture vessels, medium types, culture duration, and the morphological features under a phase contrast scope. **(B–D)** Characteristic antigenic expression in different stages of neural differentiation, Sox1 in neuroepithelia at day 7 **(B)**, Ascl1 in the dorsal spinal dI4 progenitor **(C)** at day 15, and Ptf1a in maturing dI4 INs **(D)** at day 22. D, day; Scale bar, 50 μm.

The differentiation process includes two major steps, the specification of the region-specific spinal progenitors (dI4) in the first 3 weeks followed by their differentiation to mature spinal dI4 GABAergic INs in the next 3 weeks. The specification of spinal progenitors can be roughly subdivided into three stages, induction of neuroepithelia in the 1st week, patterning of the neuroepithelia to dorsal dI4 spinal progenitors by morphogens at the 2nd week, and expansion of the specified progenitors at the 3rd week (Figure 2A). Hence, the main difference in generating dI4 is the stage of patterning the region-specific progenitors in which a different set of morphogens is required. The characteristics of the *in vitro* generated neurons are ascertained by comparing them to those in the developing human spinal cord. To ensure reproducibility, we replicated the protocols in two cell lines hESCs (H9, line WA09) and iPSCs (IMR-90 cell lines, WiCell).

**Induction of Neuroepithelia (stage 1)**: The first step is to induce the neuroectoderm fate by using inhibitors of the BMP and TGFβ signaling, DMH1, and SB. Since the type of INs is spinal identity, we decided to include a small molecule to activate the WNT pathway to induce the caudal (hindbrain and spinal cord) neuroepithelia. This step is common to the generation of dI4 GABAergic INs differentiation (Figure 2A).

**Patterning of dI4 Spinal Progenitors (Stage 2)**: The key step in directing the differentiation of neural subtypes is the precise patterning of region-specific progenitors. The dI4 INs originate from the mid-dorsal spinal cord (dp4 domain). Hence, we use retinoic acid (RA) which ventralizes cells up to the mid-dorsal region. To minimize or avoid the generation of cell types ventral to the dp4 domain, we combine RA with cyclopamine to antagonize the ventralization effect of RA. Such a combination of opposing morphogens will limit the cells to the dp4 domain.

**Expansion and Enrichment of dI4 Spinal Progenitors (Stage 3)**: The specified neural progenitors are proliferative, which offers an opportunity to expand the population. The neural differentiation process induced by small molecules is often accompanied by a small population of non-neural and neural crest derivatives. These cells are generally more adhesive than the neural progenitors. Therefore, we build in a suspension culture step to allow neural progenitors to divide into free-floating neurospheres and non-neural cells to adhere to the bottom, thus enriching and expanding the target cell populations.

**Differentiation to Mature dI4 INs (Stage 4)**: The enriched spinal progenitors in the neurospheres are dissociated and differentiated to post-mitotic neurons in the presence of neurotrophic factors. The identity of the dI4 INs is defined by their expression of characteristic transcription factors and neurotransmitter phenotypes.

### Procedure

#### hPSC Culture Setup (Before D0)

hESCs (H9, lineWA09, passages 20–40) or iPSCs were cultured in a 6-well plate covered with irradiated mouse embryonic fibroblast (MEFs) feeder layer. **Critical Step**: hPSCs should be regularly authenticated and checked for mycoplasma contamination. The culture medium was changed daily with the hPSC medium and 20 ng/ml bFGF. Cells were passaged with Dispase II as described in the standard protocol^1^.

#### Passaging the hPSCs • Timing 1 d (D0)

(1) For thawing hPSCs, the hPSC medium is pre-warmed and 1 μl of rock inhibitor is added to 10 ml of the medium. Take the cryopreserved hPSCs out from liquid nitrogen and thaw the hPSCs by shaking in a 37°C water bath. The thawed hPSC suspension is transferred to a 15-ml centrifuge tube filled with 5 ml of hPSC medium and centrifuged for 5 min at 1,000 rpm. The supernatant is discarded, and 3 ml culture medium is added to the centrifuge tube. The cells are inoculated into 6-well plates that were coated with MEF at a density of 90,000 cells/ml per well. Avoid too high or low density of cells; excessive cell density inhibits neural induction, leading to a less-efficient production of neurons, whereas low cell density decreases cell survival and proliferation. General maintenance of hPSC cultures requires daily removal of spent media and replenishment with fresh hPSC medium with bFGF. The daily microscopic observation showing clear boundaries, translucent cytoplasm, and uniform morphology indicates that the cell state is appropriate. **Critical Step**: Ensure that the cells are undifferentiated and uniform in morphology. Partially differentiated cells can reduce the probability of synchronous differentiation of cells into neuroepithelial cells.
(2) To prepare for differentiation, we maintain hPSCs in a 6-well plate containing MEFs (prepared one day in advance) with 2.5 ml of hPSC medium containing 20 ng/mlofbFGF. When the culture becomes 80% confluent (4–7 days), aspirate the old culture medium and add 1 ml of pre-warmed dispase (1 mg/ml) to each well. Then the 6-well plate is placed in the incubator at 37°C 5% CO_2_ for 3–5 min
(3) When the edge of the hPSC colonies starts to curl, aspirate off the digestive enzyme dispase. Gently rinse hPSCs with 2 ml of DMEM/F12 two times without interfering with loose hPSC colonies.
(4) Add 2 ml of fresh hPSC medium to each well. Slowly rotate the 6-well plate, blow the colonies with a 1-milliliter pipette, and triturate the large colonies into a small cell mass of 50–100 μm. **CRITICAL STEP**: Breaking down the hPSC colonies into too-small fragments will result in a lower neural differentiation rate. Avoid pipetting hPSC colonies more than five times.
(5) The hPSC colonies are collected into a new 15-ml centrifuge tube and centrifuged at 1,000 rpm for 2.5 min. After discarding the supernatant, the cells are resuspended in 1.5 ml hPSCs medium and incubated overnight in a 37°C incubator. The neural differentiation will begin on the 2nd day of hPSC culture.

#### Differentiation Stage 1: Induction of Neuroepithelial Cells (NEPs) From hPSCs • Timing 7 d (D1–D7)

(6) For preparing 50 ml of neural induction medium (NIM) include 24.5 ml of DMEM/F-12, 24.5 ml of neurobasal medium, 0.5 ml of NEAA, and 0.5 ml of N-2 supplement. One day after passaging the hPSCs, the original hPSC medium was changed to 2 ml NIM with the presence of SB431542 (2 mM), DMH1 (2 mM), and CHIR99021 (3 mM). This is regarded as day-1 (D-1) of differentiation. The morphology of the cells is observed every day and the medium changed every other day for 7 days.
(7) On day 7 (D7) of differentiation, special tubular epithelial-like structures were observed under the microscope (Figure 2A). This is indicative of the generation of NEPs.

#### Differentiation Stage 2: Patterning NEPs to dI4 Spinal Precursors • Timing 7 d (D8–D14)

(8) The day before this stage, a 6-well plate with MEF is prepared and incubated overnight in a 37°C incubator.
(9) Right before patterning progenitors, prepare the neural specification medium for dI4 INs. For 100 ml, add 98 ml NIM, 2 ml of 50× B27 additive. Then add small molecules (1 μM CHIR99021, 2 μM SB431542, 2 μM DMH1, 0.1 μM RA, and 0.5 μM CYC) for dI4 IN culture.
(10) Add 1 mg/ml dispase solution to each well of the NEP cultures and digest for 3 min in the incubator.
(11) When the colony edges curl, the dispase solution is aspirated and the culture washed twice with pre-warmed DMEM/F12 solution, 2 ml per pass.
(12) Add 2 ml of DMEM/F12 medium to each well and gently blow the cell colonies. The cell clusters are then collected to a 15 ml centrifuge tube, and centrifuged at 800 rpm for 2 min.
(13) The supernatant was discarded and the cell pellets are resuspended in the neural specification medium for dI4 neuronal cultures. The cells collected from one plate are usually replated to six plates, i.e., a 1:6 split. Inoculate 2 ml cell suspension per well and cultured for 7 days with medium change every other day.
(14) During the period, the epithelium gradually organizes into rosettes. By day 14 (D14) of differentiation, multiple layers of cells pile up in the rosette with a lumen in the center under the microscope. There are numerous rosettes in each colony. The dI4 progenitors exhibit a similar rosette structure (Figure 2A). At this stage, the MEF gradually died and are washed away during medium change.

#### Differentiation Stage 3: Expansion of dI4 Spinal Progenitor Cells • Timing 7 d (D15–D21)

(15) Prepare the expansion medium for dI4 IN progenitors. For 100 ml medium, add 98 ml NIM, 2 ml of 50× B27 supplement. Then add a small molecule (0.1 μM RA, 0.1 μM CYC) for dI4 progenitors.
(16) The multi-layered neural rosettes are often loosely attached to the plate by D14 and can be readily dislodged mechanically. Add 2 ml DMEM/F12 per well and gently blow off the colonies. This step often leaves some of the adherent cells surrounding the rosettes in the dish.
(17) The dislodged colonies are collected into a 15-ml conical tube, centrifuge at 800 rpm for 2 min, and resuspend in the expansion medium for dI4 progenitors, respectively. The cell suspensions are transferred to T25 flasks (8 ml/flask) and cultured for 7 days. Cells from every 2 to 3 wells can be collected into a T25 flask.
(18) The neural progenitors in suspension cultures grow as neurospheres (Figure 2A). The culture medium is changed every other day by standing the flask to let the spheres settle down to the bottom (2–3 min) and gently aspirating the supernate followed by replenishing the volume with a fresh medium. **Critical Step**: In the first few days, there may be cells adhering to the flask. The non-neural cells are often flat and adhere to the plate tightly. By swirling the flask gently, the loosely attached neural progenitors and their clusters (spheres), but not the non-neural cells, are dislodged. The dislodged cells are transferred to a new flask and the old flask is discarded. This step enriches the neural progenitors by removing the adherent non-neural cells, it prevents the neurospheres from attachment. **Pause Point**: The **Spinal Cord Progenitor Cells** may be cryopreserved at **D15-D21**. Two days before cell freezing, treat progenitor clusters with accutase solution into small spheres (20–50 μm). Spin down the spheres at 800 rpm for 2 min and resuspend in 1 ml of cryopreservation medium that contains 10% (vol/vol) DMSO, 30% (vol/vol) FBS and 60% (vol/vol) NIM. Place the cryopreservation vial in a cryopreservation container; condition the container at −80°C overnight before transferring the vials to a liquid nitrogen tank.

#### Differentiation Stage 4: Differentiation of Spinal Progenitors to dI4 INs • Timing 21 d (D22–D42)

(19) Preparation of neural differentiation media and coverslips. One day before neuronal differentiation, coverslips (13-mm) in 24-well plates are coated with matrigel. Dilute matrigel in DMEM/F-12 (1:50). Distribute 80 μl per well of diluted matrigel onto each coverslip in 24-well plates. Right before neuronal differentiation, prepare the neural differentiation medium (NDM) that is common for dI4 neurons. For 50 ml, include 48 ml of neurobasal medium, 0.5 ml of NEAA, and 0.5 ml of N-2 supplement. Then add 1 ml of 50× B27 supplement, 50 μl AA at (final concentration) 1 mM, 50 μl cAMP at 1 mM, 50 μl of Brain-Derived Neurotrophic Factor (BDNF) at 10 ng/ml, 50 μl of Glial cell-derived neurotrophic factor (GDNF) at 10 ng/ml.
(20) On day 22, collect the dI4 neurospheres into two separate 15-ml centrifuge tubes and centrifuged for 3 min, at 800 rpm. Following aspiration of the supernatant, add 1 ml of pre-warmed accutase solution, re-suspend the neurospheres and incubate the tube at 37°C. **Critical Step**: Shake the tube periodically and observe the spheres every 3 min until the spheres become fuzzy.
(21) Add 1 ml DMEM/F-12 and centrifuge the tubes at 1,000 rpm for 2.5 min. Discard the supernatant and resuspend the cell pellet with 1 ml of NDM for dI4 neurons. Count the cells and adjust the cell concentration to 500,000 live cells/ml.
(22) Plate the neural progenitors onto the matrigel-coated coverslips by adding 50 μl (10,000 cells) cell suspension onto each coverslip. **Critical Step**: Do not let the medium spread out of the edge of the coverslip to avoid the adherence of cells outside of the coverslip. The cell density may be adjusted depending on the need of the culture. In general, do not plate progenitors at a density higher than 10,000 cells per coverslip as a higher density inhibits progenitors from differentiation and maturation.
(23) After culturing at 37°C and 5% CO_2_ for 2 h, the neural progenitors attach to the coverslip. Add 300 μl of NDM to each well. **Critical Step**: Add the medium along the wall of the well slowly to avoid dislodging the loosely adhered cells.
(24) The medium for the neuronal differentiation cultures is changed twice a week by removing half of the medium and replenishing it with the same volume of the fresh medium. The coverslip cultures may be assayed at any time point. We assay the cultures at 1 week and 3 weeks post-plating.

#### Characterization of the Differentiating Cells by Immunofluorescence Staining • Timing 2 d (D7, D14, D22, D28, D42)

(25) At desired endpoints of the differentiation, cells grown on coverslips are rinsed with PBS briefly and fixed with 500 μl per well of 4% (wt/vol) paraformaldehyde (PFA) for 30 min at room temperature. This step is operated in a fume hood.
(26) Remove and safely dispose of the PFA. Wash three times with 500 μl of PBS per well. **Pause Point**: You can fill each well with 1 ml of PBS, wrap the plate with parafilm, and store it at 4°C for 2 weeks before staining.
(27) Pick out the coverslip and place it on a stage. Block for nonspecific binding by adding 50 μl of QuickBlock^TM^ Blocking Bufferto each coverslip. Incubate at room temperature for 1 h.
(28) Remove blocking solution. Prepare QuickBlock^TM^ Primary Antibody Dilution Buffer and add the proper antibody concentrations as listed in Table 1. Add 50 μl onto each coverslip and incubate at 4°C overnight.
(29) Wash three times with 100 μl of PBS for 15 min each at room temperature and add 50 μl of secondary antibody (prepared in QuickBlock^TM^ Secondary Antibody Dilution Buffer) to each coverslip for 1 h at room temperature.
(30) Wash three times with 100 μl of PBS for 15 min. Add 1:1,000 DAPI to each well for 5 min at room temperature. Protect the plate from light. Wash three times with 100 μl of PBS for 15 min each at room temperature. Mount the coverslips and seal the glass slide with Fluoromount-G^®^.
(31) Images are collected using an Olympus FV3000 fluorescence laser-scanning confocal microscope (Shinjuku Monolith, 2-3-1 Nishi-Shin-juku, Shinjuku-ku, Tokyo 163-0914, Japan). ImageJ is used to merge color channels and generate images for publication.

### Timing

Steps 1–5, passaging, seeding hPSCs for differentiation: 1 day.

Steps 6–7, induction of neuroepithelial cells from hPSCs: 7 days.

Steps 8–14, patterning spinal progenitor cells: 7 days.

Steps 15–18, expanding spinal progenitor cells: 7 days.

Steps19–24, differentiation to spinal dI4 INs: 21 days.

Steps 25–31, immunofluorescence staining of cells at desired stages of differentiation: 2 days (D7, D14, D22, D28, D42).

### Immunofluorescence Staining of Cells

On the D7, D14, D22, D28, and D42 of differentiation, coverslip cultures were washed once with phosphate buffer saline (PBS) and fixed with 500 μl of 4% paraformaldehyde (PFA) per well for 30 min. Remove and safely dispose of the PFA. Wash three times with 500 μl of PBS per well. Block for nonspecific binding by adding 500 μl of QuickBlock^TM^ Blocking Buffer to each well and incubate at room temperature for 1 h. Pick out the coverslip with a pair of fine forceps and place it on a stage. Add 50–100 μl of antibody solution to each coverslip and incubate at 4°C overnight. Wash three times with 100 μl of PBS for 15 min each at room temperature before incubation with secondary antibody in a light-tight box for 1 h. Wash three times with 100 μl of PBS for 15 min each and add DAPI solution to each coverslip for 5 min. The stained coverslips were washed with PBS three times before mounted onto glass slides and sealed for microscopic observation. The population of positive expressing cells among total differentiated cells (DAPI-labeled) was counted using the ImageJ software. The immunofluorescence staining data were obtained from at least three biological replicates and data were replicated three times in two different cell lines (H9 and IMR-90).

### Immunofluorescence Staining of Embryonic Spinal Cord

Human fetal spinal cord tissues (12-week) were obtained from Tongji Hospital, Tongji Medical College, Huazhong University of Science and Technology. The specimens were rinsed with sterile saline and fixed in PFA at 4°C for 24 h. After rinsing with PBS, the spinal cord tissue was cryoprotected in 20% and 30% (wt/vol) sucrose solutions in succession. After the spinal cord tissues completely sunk in the 30% sucrose, the samples were cut into 30-μm sections using a cryostat. The sections were washed in PBS carefully and permeabilized with 0.5% (vol/vol) Triton X-100 for 30 min, then incubated in 3% BSA to block nonspecific binding for 1 h. The slices were incubated with primary antibodies at 4°C overnight. After three washes in PBS, the slices were incubated with secondary antibodies for 1.5 h at room temperature. DAPI was used to stain the nuclei. The images were captured employing a confocal microscope (Olympus, FV 3000).

## Results

We arbitrarily divide the spinal IN differentiation process into four stages even though it is a continuous process. Troubleshooting advice can be found in Table 2. The first stage is the induction of NEPs in the presence of small molecules SB431542 and DMH1 in the first week. Since dI4 neurons are from the spinal cord, we also add CHIR99021 to caudalize the differentiating neuroepithelia at the first week. Under such a condition, the vast majority of the differentiated cells become neuroepithelia. Morphologically, they display columnar cells that often line up in a tubular fashion and express the neuroepithelial marker Sox1 (Figure 2B).

**Figure 3:**
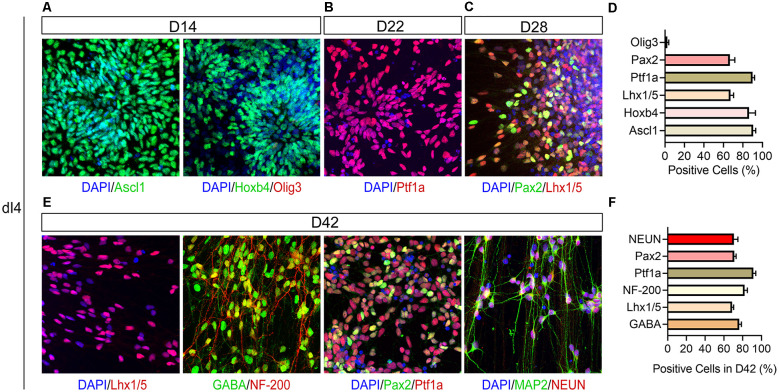
Immunophenotyping of hPSC-derived dI4 INs. **(A)** Confocal images showing expression of Ascl1, Hoxb4, and Olig3 at D14; Ptf1a **(B)** at D22, Pax2 and Lhx1/5 **(C)** in dI4 neural progenitors at D28. **(D)** The percentage of positive cells in **(A–C)**. **(E)** Immunostaining of differentiated dI4 mature neurons for GABA, NF-200, Pax2, Lhx1/5, NEUN, and MAP2 at D42. **(F)** The percentage of positive cells in **(E)**. Scale bar, 50 μm. Data are presented as mean ± SEM of three independent experiments.

The 2nd stage (D8–14) is to pattern the differentiating precursors to either the dorsal dI4 spinal progenitor fate. In the presence of RA and CYC as well as CHIR, the differentiating neural progenitors divide and form multi-layered rosettes which we call neural tube-like rosettes. They also acquire a dorsal spinal fate by expressing Hoxb4 (86.3%), a spinal transcription factor, and Ascl1 (90.7%), a dI4 associated transcription factor (Figures 2C, 3A,D). These cells almost do not express Olig3, a ventral transcription factor expressed in dI1–3, V0, V2, V3 spinal progenitors (Lai et al., 2016; Figures 3A,D). These results further confirm that our progenitors are limited to the dI4 progenitor fate. About 90% of the differentiated cells expressed Ascl1 and Hoxb4.

**Table 2:** Troubleshooting table.

**Step**	**Problem**	**Possible reason**	**Solution**
1	Low cell survival post-thaw.	Cell death during thaw.	Do not let the vial of cells completely thaw in the water bath.
2	#x02013;5	hPSCs are not reaching confluency at the predicted time.	hPSCs are too big or too small; they contain too many dead cells.	The passage ratio can be changed to increase cell density. Gently pipette the iPSCs during passage and medium changes.
18	The medium becomes yellow too quickly.	Too many cells in one flask.	Cells should be fed every day or split into two flasks. An acidic environment may increase cell death.
20	Large spheres.	Cell proliferation.	Extend the processing time of Accutase Solution.
21	#x02013;23	Cell layers are detaching from plate during differentiation.	Inefficient Matrigel coating.	Use fresh Matrigel and make sure to coat for 24 h. Take out the Matrigel-coated tissue culture plates from the freezer. Leave the plate at 37°C in an incubator for 1 h.
24	#x02013;31	Cell layer peeling during staining.	Cell layers are releasing during fixation and wash steps.	Fix for 1 h instead of 30 min. During wash steps, a transfer pipette may be used to carefully exchange liquids.

At stage 3, the patterned neural progenitors are cultured in suspension as neurospheres. The purpose of suspension culture is to expand and enrich the progenitors. Quantification of the cells plated from the day-21 dI4 neurospheres (assayed at day-22) indicated that 90% of cells were Ptf1a^+^ (Figures 2D, 3B,D).

**Figure 4:**
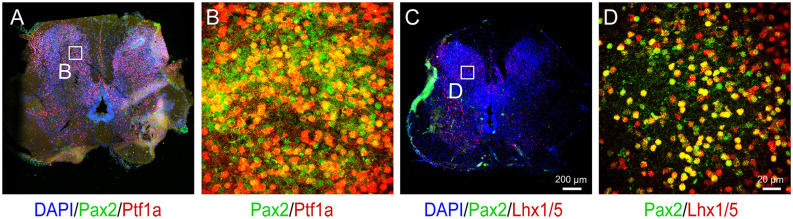
Immunophenotyping of 12-week human embryo spinal cord. **(A)** Immunostaining for Pax2 and Ptf1a in the spinal cord. Panel **(B)** is the magnified view on the inset in **(A)**. **(C)** Immunostaining for Pax2 and Lhx1/5 in the spinal cord. Panel **(D)** is the magnified view on the inset in **(C)**. Scale bar, 200 μm **(A,C)** and 20 μm **(B,D)**.

Once the dI4 progenitors are specified, the last step is to differentiate them in adherent culture to mature neurons. The dI4 neurons express Pax2 and Lhx1/5 besides Ptf1a (Glasgow et al., 2005). Indeed, 66.6% and 67.3% cells were Pax2^+^ and Lhx1/5^+^ at D28, respectively (Figures 3C,D). By 6 weeks in culture, the cells exhibit many markers associated with mature dI4 neurons. They expressed NEUN/MAP2 (70.7%), markers of mature neurons, as well as GABA (76.3%), NF-200 (82%), Pax2^+^ (71%), and Lhx1/5 ^+^ (68.7%) at D42, indicating that the majority of cells become spinal dI4 GABAergic INs (Figures 3E,F). Immunostaining for these transcription factors in the embryonic tissues validates the specificity and pattern of expression for Pax2, Lhx1/5, Ptf1a (Figures 4A–D). Therefore, our hPSC-derived dI4 progenitors resemble those in the developing spinal cord.

## Discussion

### Development of the Protocol

The protocols were devised based on the principles of dI4 neuron development learned from animals and validated in the developing human spinal cord. They were then optimized by using small molecules and minimizing the involvement of peptide growth factors to enhance reproducibility across laboratories. The protocols described here were reproduced in multiple hPSC lines.

The neural progenitor identities in the vertebrate spinal cord are specified along the rostral-caudal and dorsal-ventral axes. In addition, there is a temporal influence of development on these spatial coordinates such that distinct cell fates emerge at different times during development. This yields a four-dimensional system for establishing spinal neuron cell fate (Lu et al., 2015). The rostral-caudal positional identities are coordinated by opposing gradients of fibroblast growth factor (Mazzoni et al., 2013; Maury et al., 2015; FGF, rostralizing) and retinoic acid (RA)/WNTs (caudalizing). The dorsal-ventral axis is governed by ventralizing SHH (Lee et al., 2007; Li et al., 2008; Patani et al., 2011) produced by the floorplate, and dorsalizing signals from the roof plate such as BMPs and WNTs (which are members of the Wingless^+^ family). The use of opposing sets of morphogens to generate enriched neural subtypes has been proposed (Tao and Zhang, 2016) and demonstrated in generating spinal motor neurons (Du et al., 2015). dI4 neurons are from the spinal cord. We hence used RA and a WNT agonist (CHIR99021) to pattern the neuralized progenitors to the spinal fate. The dI4 neurons derive from the mid-dorsal dp4 domain for which one would dorsalize the neural progenitors. Interestingly, RA tends to pattern neural progenitors to the ventral regions up to the mid-dorsal part (dp4). Since a single morphogen generates a gradient that induces a spectrum of ventral to dI4 progenitors, we blocked the ventralization effect of RA by using an SHH inhibitor, cyclopamine (CYC) so as to limit the progenitors to the dI4 identity. Following the withdrawal of morphogens, the spinal neural progenitors were further differentiated to region-specific neurons.

### Applications of the Protocol

Spinal INs form complex networks in the spinal cord and regulate neural functions under physiological and pathological conditions, including circuit regulation, neuropathic pain, spasticity, and autonomic dysreflexia. The ability to generate enriched human spinal IN subtypes enables the analysis of cellular, molecular, and functional attributes of the human cells. Since hPSCs are readily modified genetically, spinal INs from the hPSCs may be readily engineered to establish platforms for screening or testing compounds that regulate IN functions (Yang et al., 2021).

The role of INs in both healthy and injured spinal cords is of high interest (Zholudeva et al., 2021). The enriched populations of dI4 GABAergic INs may also be transplanted into the spinal cord to examine how they integrate within a native tissue setting and whether they bear therapeutic potential in the injured spinal cord. Inhibitory INs converge on spinal motor neurons to balance the neuronal excitability, hence muscle tone or spasticity. One of the inhibitory inputs comes from the proprioceptive sensory neurons in the dorsal root ganglia, whereas in the spinal cord the main inhibitory inputs are from dI4 GABAergic Ins (Jankowska, 1992). We have shown that the dI4 IN progenitors, transplanted into injured rat spinal cord, mature and form synapses with glutamatergic INs and motor neurons, mitigating spasticity (Gong et al., 2021). Therefore, the establishment of protocols for efficient differentiation from hPSCs and the availability of spinal dI4 GABAergic INs open a new avenue for treating injury and diseases of the spinal cord.

The step-by-step protocol described above yields an enriched population of spinal dI4 GABAergic INs from hPSCs. This protocol is reproduced in both ESCs and iPSCs, suggesting that it is likely applicable to other hPSCs. The stem cell-derived spinal INs subtypes may be useful in the study of human spinal IN subtype development, the establishment of drug testing platforms, and the development of novel regenerative therapies for traumatic injuries to the spinal cord.

## Data Availability Statement

The original contributions presented in the study are included in the article, further inquiries can be directed to the corresponding author.

## Ethics Statement

This study was reviewed and approved by the Medical Ethics Committee of Tongji Hospital, Tongji Medical College of Huazhong University of Science and Technology. Written informed consent was obtained from the individual(s) for the publication of any potentially identifiable images or data included in this article.

## Author Contributions

JX and HC conceived the study. JX, L-JH, ZF, H-ML, Y-QC, and Y-JL performed the experiments. JX wrote the manuscript. HC supervised the project. All authors contributed to the article and approved the submitted version.

## Conflict of Interest

The authors declare that the research was conducted in the absence of any commercial or financial relationships that could be construed as a potential conflict of interest.

## Publisher’s Note

All claims expressed in this article are solely those of the authors and do not necessarily represent those of their affiliated organizations, or those of the publisher, the editors and the reviewers. Any product that may be evaluated in this article, or claim that may be made by its manufacturer, is not guaranteed or endorsed by the publisher.

## Footnotes

^1^ http://www.wicell.org
